# Global diastolic strain rate for the assessment of left ventricular diastolic dysfunction in young peritoneal dialysis patients: a case control study

**DOI:** 10.1186/s12882-020-01742-8

**Published:** 2020-03-10

**Authors:** Jing Zhu, Fei Shi, Tao You, Chao Tang, Jianchang Chen

**Affiliations:** Department of Cardiology, The Second Affiliated Hospital of Soochow University, 1055 Sanxiang Road, Suzhou, 215004 Jiangsu Province China

**Keywords:** Strain rate, Diastolic dysfunction, Young peritoneal dialysis patients, Two-dimensional speckle tracking imaging

## Abstract

**Background:**

Left ventricular (LV) myocardial longitudinal diastolic strain rate measured by two-dimensional speckle tracking imaging (2D-STI) was proved to have a better correlation with the LV diastolic function. We aimed to use this sensitive tool to predict LV myocardial diastolic dysfunction in young peritoneal dialysis (PD) patients with preserved LV ejection fraction (LVEF).

**Methods:**

We enrolled 30 PD patients aged ≤60 with LVEF ≥54% and classified as normal LV diastolic function by conventional echocardiography, and 30 age- and sex-matched healthy people as the control group. The left atrial maximum volume index (LAVI), LV mass index (LVMI), LVEF, LV posterior wall thickness (LVPWT), interventricular septal thickness (IVST), peak velocity of tricuspid regurgitation (TR), peak early diastolic velocity/late diastolic velocity (by Pulsed Doppler) (E/A) and E/peak velocity of the early diastolic wave (by Pulsed-wave tissue Doppler) (E/e’) were recorded by conventional echocardiographic. Next, the average LV global longitudinal systolic strain (GLS avg) and the average LV global longitudinal diastolic strain rate (DSr avg) during early diastole (DSrE avg), late diastole (DSrA avg) and isovolumic relaxation period (DSrIVR avg) were obtained from 2D-STI. Combined them with E, the new noninvasive indexes (E/DSrE avg., E/DSrA avg. and E/DSrIVR avg) were derived.

**Results:**

The PD group ‘s LVEF, E/e′, TR and LAVI were in the normal range compared with the controls, and only e′ (*p* < 0.001) was decreased. The LVMI (*p* < 0.001), LVPWT (*p* < 0.001), IVST (*p* < 0.001) increased while E/A (*p* < 0.001) decreased. The GLS avg. (*p* = 0.008) was significantly decreased in PD patients compared with the controls. DSrA avg. (*p* = 0.006) and E/DSrE avg. (*p* = 0.006) were increased, while DSrE avg. (*p* < 0.001), DSrIVR avg. (*p* = 0.017) and E/DSrA avg. (*p* < 0.001) decreased. After the multivariable regression analysis, the correlation between DSrE and the conventional parameters including LVPWT (*p* < 0.001), E/A (*p* < 0.001) still remained significant.

**Conclusions:**

Young PD patients with preserved LVEF already exhibited myocardial diastolic dysfunction. Global diastolic strain rate indexes were valuable parameters to evaluate diastolic dysfunction. Additionally, LVPWT was highly correlated with DSrE, such parameter should be taken into account for predicting the early LV diastolic dysfunction in clinical practice.

## Background

Currently, more young people are developing chronic kidney disease (CKD), adding to the burden on families and countries. Peritoneal dialysis (PD) is advantageous because it preserves residual renal function and is inexpensive, with greater maneuverability outside the hospital, PD is becoming a popular choice for renal replacement therapy in young uremic patients, especially in developing countries. In the early stage of CKD, cardiac structure and function already begin to change and gradually aggravate in patients with deteriorated renal function approaching or on dialysis according to some studies [1, 2]. Until now, more and more studies have focused on studying left ventricular (LV) diastolic dysfunction because diastolic dysfunction seems to occur earlier than systolic dysfunction in PD patients. And impaired LV diastolic function has been considered as the primary determinant of symptoms related to cardiovascular events, and therefore, the incidence of sudden cardiac death, arrhythmia, and congestive heart failure tend to be higher in patients with LV diastolic dysfunction than in those without [3–5]. Thus, identifying young PD patients’ diastolic dysfunction at the early stage is of clinical importance.

Cardiac catheterization is known as the gold standard to evaluate the extent of diastolic dysfunction, but it is invasive and not easily accepted by the majority of patients. In that case, noninvasive estimation of LV diastolic dysfunction is a clinical requisite. With the development of the two-dimensional speckle tracking imaging technique (2D-STI), it is now possible to use LV global longitudinal diastolic strain rate (DSr) to measure LV diastolic dysfunction indirectly and noninvasively [6–8]. Therefore, the objective of this study was to use 2D-STI to predict LV myocardial diastolic dysfunction in the preclinical phase in young PD patients with preserved LVEF.

## Methods

We enrolled 30 young uremic patients who underwent PD catheter insertion in our PD center from September 2016 to September 2018; there was the equal distribution in age and sex. The inclusion criteria were as follows: (1) progression to end-stage renal disease, (2) underwent PD regularly for 3–18 months, (3) age ≤ 60 years in order to avoid the effect of age on diastolic function [9, 10], (4) the basic rhythm was sinus rhythm, (5) the LV ejection fraction (LVEF) was ≥54% [11], and (6) LV diastolic function was classified as normal by conventional echocardiography [10]. We excluded patients with nonsinus rhythm, congenital heart disease, primary valvular heart disease, moderate or severe mitral and aortic regurgitation, heart block, pacemaker implantation, hypertensive heart disease, having had acute myocardial infarction or acute heart failure, regional wall motion abnormality detected by echocardiography, any other nonrenal heart diseases, moderate and severe anemia, and unsatisfactory echocardiographic images. Furthermore, 30 age- and sex-matched healthy people were chosen for the normal control group, who were free of heart and kidney disease by confirmation with electrocardiogram, echocardiography, routine blood and urine examination, and renal function (normal serum creatinine (CREA) and blood urea nitrogen (BUN)).

Firstly, some baseline characteristics of the study subjects were collected, including age, sex, body mass index (BMI), body surface area (BSA), blood pressure, peritoneal dialysis time, CREA, BUN, hemoglobin (Hb), calcium (Ca), phosphorus (P), and the causes of end stage renal disease (ESRD). Then, studies were performed using a Model GE Vivid E9 ultrasound system with an M5S phased array transducer using a transmission frequency of 2.0–4.5 MHz. Images were taken of the parasternal view with patients lying in the left decubitus position. Using M-mode echocardiography, we recorded the LV internal diameter at end-diastole (LVIDD), LV internal diameter at end-systole (LVIDS), interventricular septal thickness (IVST), and LV posterior wall thickness (LVPWT). The LVEF was calculated using a modified Simpson’s biplane method, and the LA maximum volume (LAVmax) was obtained just before mitral valve opening using an area-length method from the apical 4- and 2-chamber views. All cardiac chamber volumes and diameter were indexed to the body surface area (BSA), and the normal systolic function was defined as an LVEF ≥54% [11].

Then, we calculated LV mass: LV mass (g) = 0.8 × 1.04 × [(LVIDD + LVPWT + IVST)^3^ - LVIDD^3^] + 0.6 [11], and defined the LV mass index (LVMI) as the LV mass/height^2.7^ [12]. The peak early diastolic velocity (E), late diastolic velocity (A) of the mitral orifice and E deceleration time (DT) of the E-wave were measured by pulsed Doppler from the apical 4-chamber view. Then, the E/A ratio was calculated. The peak velocity of the early (e′) and late (a′) diastolic wave were measured by pulsed-wave tissue Doppler, with the sample volume close to the mitral valve annulus in the apical 4-chamber view in the lateral wall. The peak velocity of tricuspid regurgitation (TR) was achieved in the apical four-chamber view with continuous-wave Doppler. We calculated the E/e′, and defined e’ < 10 cm/s, E/e’ > 13, TR > 2.8 m/s and LAVI> 34 mL/m^2^ as the recommended variables for identifying diastolic dysfunction. If more than half of the available variables do not meet the cutoff values for identifying abnormal function, the LV diastolic function was classified as normal [10]. All echocardiographic measurements were performed by experienced echocardiographic technicians who were blinded to the clinical conditions.

**Fig. 1:**
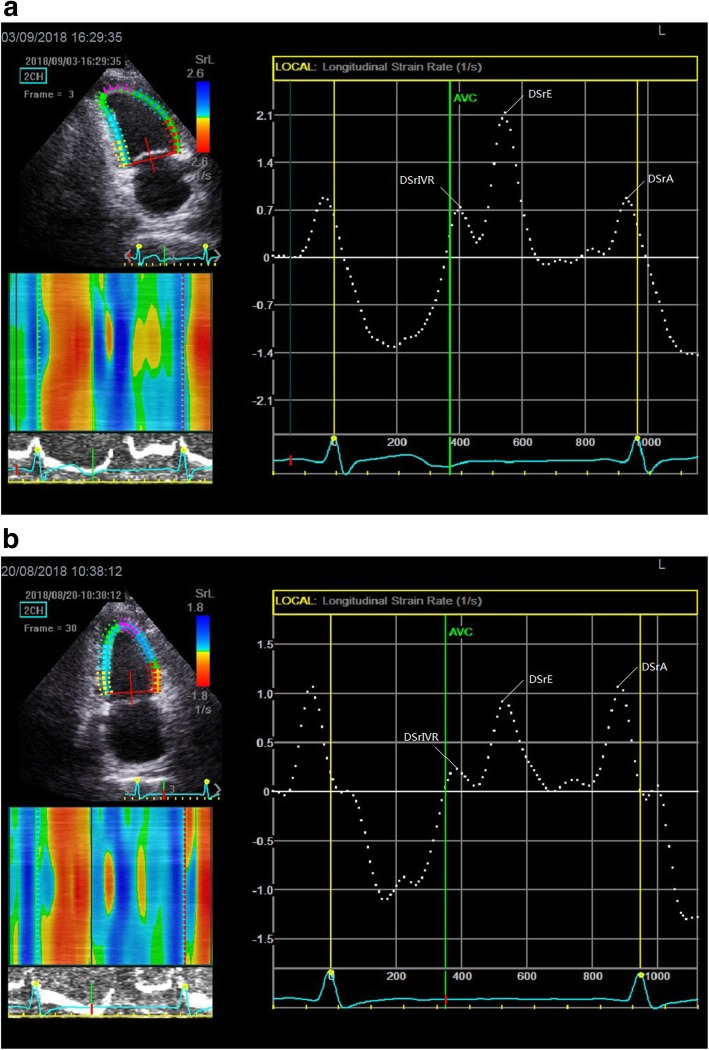
Left ventricular longitudinal diastolic strain rate curves during early diastole (DSrE), late diastole (DSrA) and the isovolumic relaxation period (DSrIVR) obtained from the 2-chamber views. (a was the control group and b was the PD group)

Next, dynamic 2D sonographic images of 3 cardiac cycles were obtained from the standard apical long-axis view, 4- and 2-chamber views. These images were digitally stored on hard disks for myocardial strain analysis using offline software EchoPAC 201. Manual tracings of the endocardial border during end-systole in 3 apical views were performed, and when the system automatically determined the tracking quality for each analyzed segment, the average of global longitudinal systolic strain (GLS avg) was automatically obtained. Then, from the LV longitudinal diastolic strain rate curves for three views of the LV myocardial: the LV longitudinal diastolic strain rate (DSr) during early diastole (DSrE), during late diastole (DSrA), and during the isovolumic relaxation period (DSrIVR) from the apical long-axis view, 4- and 2-chamber views (Fig. 1). We collected these parameters and calculated their average, and combined with E, the diastolic indices E/DSrIVR avg., E/DSrA avg., and E/DSrE avg. were obtained.

Lastly, variates were presented as the mean ± standard deviation, median with interquartile range, or number with percentage. Continuous variates with normal and skewed distributions were compared using the paired *t* test and Wilcoxon rank-sum test, respectively. Categorical variates were compared using the χ2 test. The correlation between two variates was determined by Pearson’s linear correlation and visualized by scatter plots. To avoid the influence of the multicollinearity between parameters, stepwise multivariate linear regression was constructed to estimate the association between DSrE and conventional echocardiography parameters. Moreover, internal validation was performed using at least 1000 bootstrap samples. Statistical analysis was performed using SPSS software (version 22, IBM, USA). A *P* value < 0.05 was considered as statistically significant.

## Results

A total of 30 patients were included, and their average PD time was 11 months. In our study, no significant differences were found in subjects’ age (*p* = 0.718), BMI (*p* = 0.353), BSA (*p* = 0.371) and Ca (*p* = 0.445). The SBP (*p* = 0.008), DBP (*p* = 0.002), CREA (*p* < 0.001), BUN (*p* < 0.001) and P (*p* < 0.001) in young PD patients were significantly increased, while Hb (*p* < 0.001) were decreased. The underlying causes of end stage renal disease (ESRD) were glomerulonephritis in 56.6%, hypertensive nephrosclerosis in 16.7%, diabetic nephropathy in 10%, polycystic kidney in 6.7%, nephrotic syndrome in 3.3%, and not identified in 6.7% of patients. Overall, 76.7% of the patients were using Ca^2+^ channel blockers, 60% were using diuretics and 63.3% were using α-receptor blockers (Table 1).

**Table 1:** The Comparison of clinical characteristics in patients and controls

Variable	Control *n* = 30	Case *n* = 30	*P*-value
Age, years	44.6 ± 10.4	44.8 ± 9.5	0.718
Men, n (%)	19 (63.3%)	19 (63.3%)	
PD time, months	–	11 (3–18)	
BMI, kg/m^2^	22.35 ± 2.39	22.94 ± 2.72	0.353
BSA, m^2^	1.655 (1.527,1.870)^a^	1.663 (1.501,1.763)	0.371
SBP, mmHg	119.6 ± 12.60	135.1 ± 26.31	0.008
DBP, mmHg	74.8 ± 7.87	84.2 ± 11.34	0.002
CREA, umol/L	60.6 ± 12.25	937.2 ± 318.33	< 0.001
BUN, mmol/L	4.9 ± 1.16	20.0 ± 6.94	< 0.001
Ca, mmol/L	2.22 ± 0.13	2.18 ± 0.22	0.445
P, mmol/L	1.11 ± 0.23	1.76 ± 0.58	< 0.001
Hb, g/L	135 ± 15.0	108 ± 13.8	< 0.001
Cause of ESRD			
Glomerulonephritis		17 (56.6%)	
Diabetic nephropathy		3 (10%)	
Hypertensive nephrosclerosis		5 (16.7%)	
Polycystic kidney		2 (6.7%)	
Nephrotic syndrome		1 (3.3%)	
Unknown		2 (6.7%)	
Medication use			
Diuretics		18 (60%)	
RAAS blockers		15 (50%)	
Ca^2+^ channel blockers		23 (76.7%)	
α-Receptor blockers		19 (63.3%)	
β-Receptor blockers		15 (50%)	

Values are presented as mean ± SD, median (interquartile range), or number of subjects (%); ^a^Median (25th, 75th percentile) was used for variables that were not distributed normally, *PD* peritoneal dialysis, *BMI* body mass index, *BSA* body surface area, *SBP* systolic blood pressure, *DBP* diastolic blood pressure, *CREA* serum creatinine, *BUN* blood urea nitrogen, *Ca* calcium, *P* phosphorus, *Hb* hemoglobin, *ESRD* end stage renal disease, *RAAS* renin-angiotensin-aldosterone system

The conventional echocardiography parameters and speckle tracking imaging parameters are shown in Table 2. PD group’s E/e’, TR and LAVI were in the normal range. The LVEF (*p* = 0.053), E/e’ (*p* = 0.262), TR (*p* = 0.465) and LA volume index (*p* = 0.209) had no significant difference compared with the controls, while e’ (*p* < 0.001) was significantly decreased. Furthermore, LVIDS/BSA (*p* = 0.049), LVMI (*p* < 0.001), LVPWT (*p* < 0.001), IVST (*p* < 0.001) increased significantly, but had reduced E (*p* < 0.001) and E/A (*p* < 0.001). And DT (*p* = 0.334), LVIDD/BSA (*p* = 0.054) had no significant difference between the groups. In strain and strain rate, GLS avg. was decreased significantly (*p* = 0.008), while it was still in the normal range. And the DSrIVR avg. (*p* = 0.017), DSrE avg. (*p* < 0.001), E/DSrE avg. (*p* = 0.006), DSrA avg. (*p* = 0.006), and E/DSrA avg. (*p* < 0.001) were significantly different from controls, while E/DSrIVR avg. (*p* = 0.644) showed no significant difference (Table 2).

**Table 2:** The Comparison of conventional echocardiography parameters and speckle tracking imaging parameters in patients and controls

Variable	Control *n* = 30	Case *n* = 30	*P*-value
LV and LA structure and function			
LVEF, %	68.14 (64.0,71.0)^a^	63.86 (58.63,70.31)	0.053
LVIDD, mm	48.75 (45.30,50.78)^a^	49.25 (45.00,53.46)	0.082
LVIDD/BSA, mm/m^2^	28.53 ± 2.72	30.01 ± 3.04	0.054
LVIDS, mm	29.94 ± 3.47	31.82 ± 5.43	0.084
LVIDS/BSA, mm/m^2^	17.87 ± 2.33	19.24 ± 3.01	0.049
IVST, mm	9.0 (8.0,10.0)^a^	10.3 (8.9,12.8)	< 0.001
LVPWT, mm	8.3 (7.98,9.70)^a^	10.1 (9.0,11.75)	< 0.001
LVMI, g/m^2.7^	35.02 ± 7.13	50.62 ± 15.67	< 0.001
LV diastolic function			
E, m/s	0.91 ± 0.22	0.69 ± 0.15	< 0.001
A, m/s	0.66 ± 0.15	0.82 ± 0.16	< 0.001
e’, cm/s	14.5 (12,17)^a^	9.0 (7.75,13.0)	< 0.001
E/A	1.3 (1.13,1.85)^a^	0.8 (0.70,0.98)	< 0.001
E/e’	6.3 (5.29,7.61)^a^	7.0 (5.25,9.04)	0.262
DT, ms	221 ± 47	233 ± 50	0.334
TR, m/s	2.2 (2.0,2.42)^a^	2.1 (1.98,2.36)	0.465
LAVI, mL/m^2^	21.84 ± 5.12	23.68 ± 6.47	0.209
Myocardial deformation indices			
DSrIVR avg., s^−1^	0.518 ± 0.194	0.398 ± 0.185	0.017
E/DSrIVR avg	1.953 (1.300,2.525)^a^	1.763 (1.291,2.618)	0.644
DSrE avg., s^−1^	1.803 (1.426,2.104)^a^	1.043 (0.823,1.509)	< 0.001
E/DSrE avg	0.487 (0.433,0.562)^a^	0.610 (0.532,0.746)	0.006
DSrA avg., s^−1^	0.925 ± 0.281	1.116 ± 0.318	0.006
E/DSrA avg	1.119 ± 0.537	0.673 ± 0.254	< 0.001
GLS avg	−22.50 ± 2.511	−20.36 ± 2.827	0.008

Values are presented as mean ± SD; ^a^Median (25th, 75th percentile) was used for variables that were not distributed normally, *LVEF* left ventricular ejection fraction, *LVIDD* left ventricular internal diameter at end-diastole, *LVIDS* left ventricular internal diameter at end-systole, *IVST* interventricular septal thickness, *LVPWT* left ventricular posterior wall thickness, *BSA* body surface area, *LVMI* left ventricular mass index, *E* peak early diastolic velocity (by Pulsed Doppler), *A* late diastolic velocity (by Pulsed Doppler), e’, peak velocity of the early diastolic wave (by Pulsed-wave tissue Doppler), *DT* deceleration time of the E-wave (by pulsed Doppler), *TR* the peak velocity of tricuspid regurgitation (by Continuous-wave Doppler), *LAVI* the LA maximum volume index, *DSrIVR* strain rate during the isovolumic relaxation, *DSrE* early diastolic strain rate, *DSrA* late diastolic strain rate, *GLS* global longitudinal systolic strain, *avg.* average

**Fig. 2:**
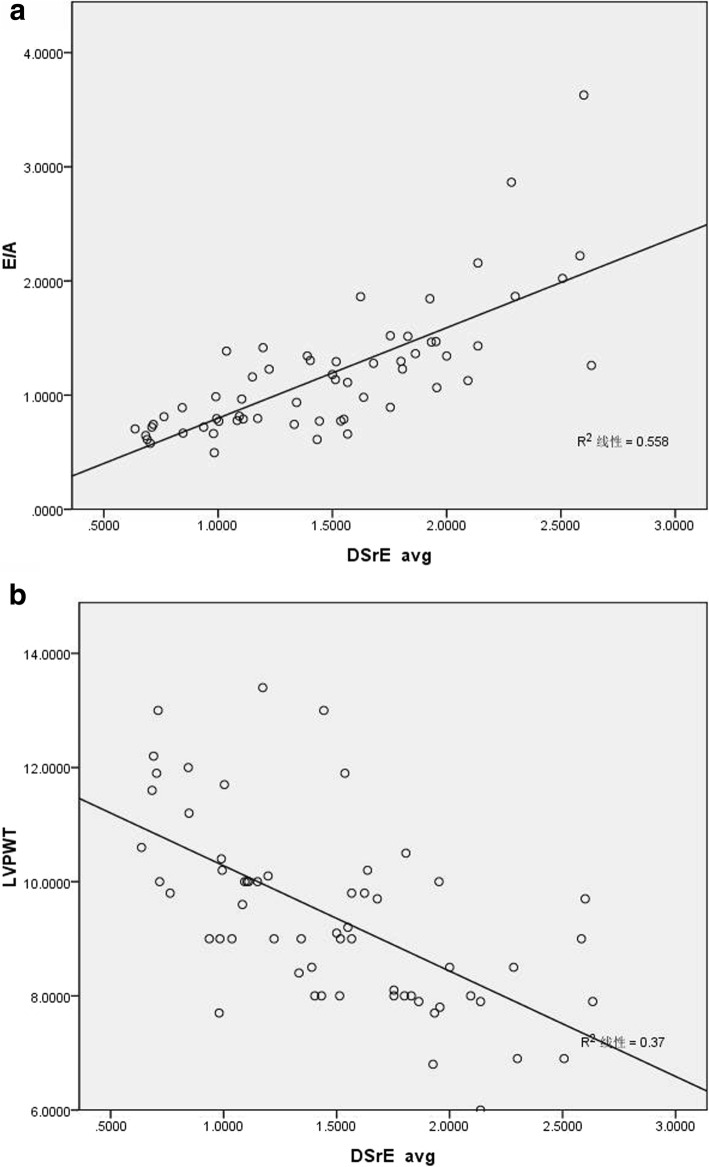
**a** showed the correlation of DSrE with E/A, and **b** showed the correlation with LVPWT

Association between DSrE and conventional echocardiography parameters are shown in Table 3 and Fig. 2. In Pearson analysis, DSrE had a strong correlation with E (r = 0.690, *p* < 0.001), e’ (r = 0.742, *p* < 0.001), E/A (r = 0.747, *p* < 0.001), and LVPWT (r = − 0.608, *p* < 0.001). And after stepwise multivariate linear regression, the correlations between DSrE and LVPWT (*p* < 0.001), E/A (*p* < 0.001) still made sense.

**Table 3:** The association between DSrE and conventional echocardiography parameters

	DSrE avg. Univariable pearson’s r	*P*	Multivariable Unstandardized β	Standardized β	*P*-value
age	−0.350	0.006			
TR	0.114	0.386			
LVEF	0.174	0.184			
E	0.690	< 0.001			
A	−0.520	< 0.001			
DT	− 0.069	0.603			
e’	0.742	< 0.001			
a’	−0.359	0.005			
E/A	0.747	< 0.001	0.562	0.596	< 0.001
E/e’	−0.278	0.031			
LVIDD	−0.333	0.009			
LVIDS	−0.261	0.044			
IVST	−0.525	< 0.001			
LVPWT	−0.608	< 0.001	−0.116	− 0.352	< 0.001
LVMI	−0.524	< 0.001			
LVIDd/BSA	−0.063	0.633			
LVIDS/BSA	−0.074	0.572			
LAVI	−0.272	0.035			

*TR* the peak velocity of tricuspid regurgitation (by Continuous-wave Doppler), *LVEF* left ventricular ejection fraction, E, peak early diastolic velocity (by Pulsed Doppler); A, late diastolic velocity (by Pulsed Doppler), *DT* deceleration time of the E-wave (by pulsed Doppler), e’, peak velocity of the early diastolic wave (by Pulsed-wave tissue Doppler), a’, peak velocity of the late diastolic wave (by Pulsed-wave tissue Doppler), *LVIDD* left ventricular internal diameter at end-diastole, *LVIDS* left ventricular internal diameter at end-systole, *IVST* interventricular septal thickness, *LVPWT* left ventricular posterior wall thickness, *LVMI* left ventricular mass index, *BSA* body surface area, *LAVI* the LA maximum volume index, *DSrE* early diastolic strain rate

**Fig. 3:**
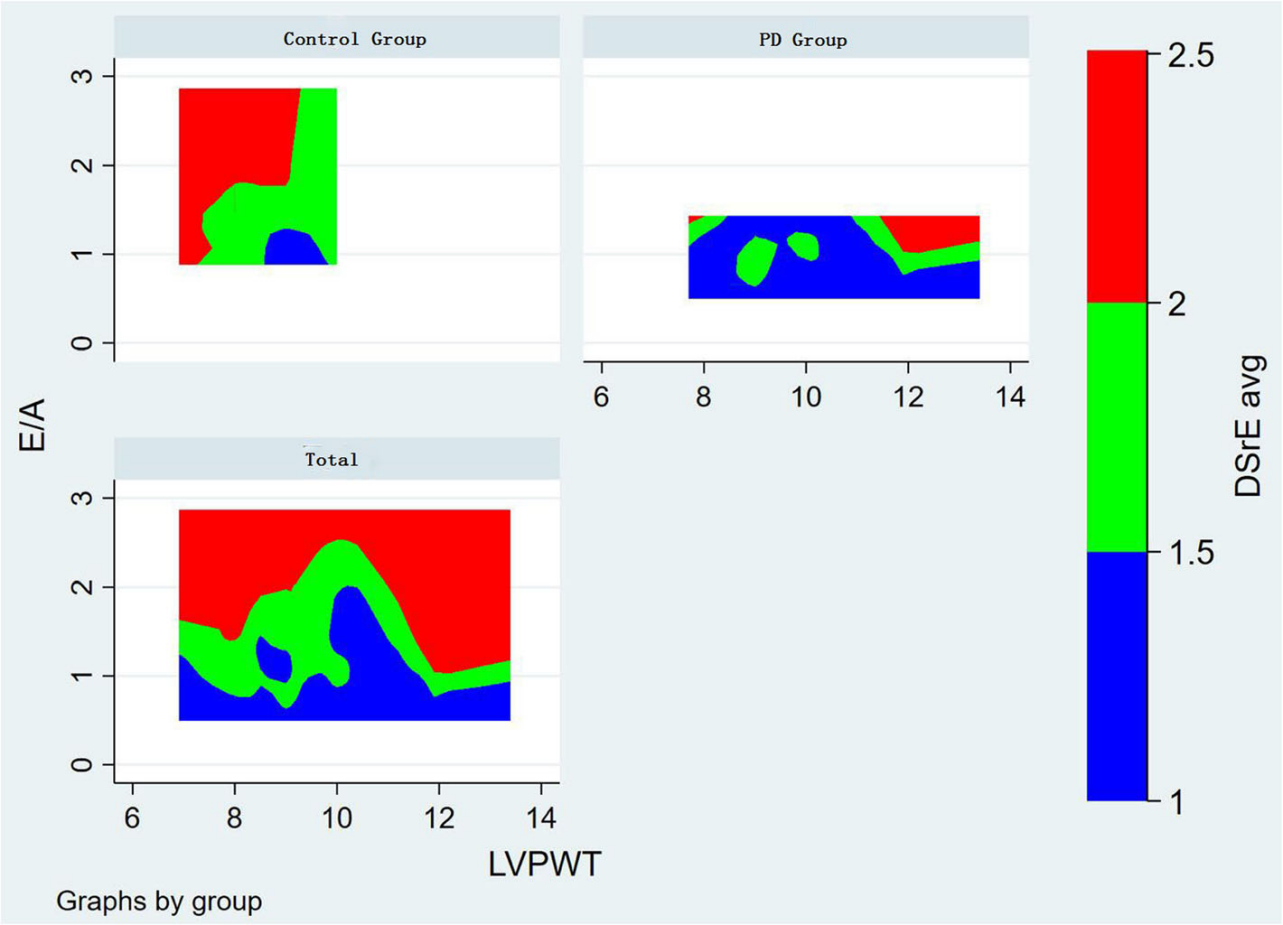
Contour plot showing the correlation among DSrE with E/A, LVPWT. The vertical axis represents E/A, and the horizontal axis represents LVPWT. The results showed that higher E/A was consistently associated with higher DSrE after controlling for the association between E/A and LVPWT. The LVPWT was higher and more diversified in PD patients than in the healthy control group. The patients showed E/A with lower level and distribution width regarding the control group

Figure 3 displays contour plot showing the association among DSrE, LVPWT and E/A. The results showed that higher E/A was consistently associated with higher DSrE after controlling for the association between E/A and LVPWT. The LVPWT was higher and more diversified in PD patients than in the healthy control group. The patients showed E/A with lower level and distribution width regarding the control group.

## Discussion

It appears that nonatherosclerotic processes, including left ventricular hypertrophy and fibrosis, account for most of the excess cardiovascular risk in uremic patients [13]. Equally, the increased pressure, volume overload, anemia, hypocalcemia, and hyperphosphatemia usually result in compensatory myocardial hypertrophy and fibrosis, making diastolic dysfunction seem to be more common and occur earlier than systolic dysfunction in young uremic patients [14–16]. Furthermore, in our previous study, we found that patients with the normal LVEF, although under long-term regular peritoneal dialysis, maintaining well-controlled blood pressure and stable ultrafiltration and adequate total fluid removal, their LV diastolic function deteriorated as the CKD progressed, in contrast to the stable LV systolic function [17]. Therefore, it seems important to detect diastolic dysfunction at the early stage of peritoneal dialysis.

According to guideline [10], our PD patients do not meet the diagnostic criteria for LV diastolic dysfunction. And considering the accuracy of e’ may be limited for several reasons such as angle, LA pressure, mitral valve disease and annular calcification, e’ cannot exactly reflect the global LV relaxation in patients with LV hypertrophy or heart failure with preserved LVEF, or in which the incidence of cardiac valve calcification is high and regional systolic dysfunction is present [6, 18–20]. Therefore, using e’ to reflect the global LV diastolic dysfunction is unsuitable. Moreover, the wall thickness of the LV ventricular such as IVST, LVPWT and LVMI in PD patients were significantly increased compared to controls, showing that the patients we studied already exhibited myocardial hypertrophy, which is a strong and independent factor for cardiovascular risk [13, 21]. Additionally, GLS is usually decreased in patients with heart failure with preserved ejection fraction, and is significantly correlated with LV end-diastolic pressure (LVEDP), showing that the decrease of GLS usually foreshadows LV diastolic dysfunction at the early stage [10]. In our study, GLS in young PD patients was in the normal range, but lower than that in the control group, indicating that LV diastolic dysfunction probably exists at the early stage of peritoneal dialysis. So we urgently needed some sensitive indicators to quantitatively assess early LV diastolic dysfunction.

LVEDP measured by cardiac catheterization and the cardiac catheterization-derived time constant of LV relaxation, (Ʈ), are important factors for evaluating the severity of diastolic dysfunction [22]. And the late gadolinium enhancement (LGE) cardiac magnetic resonance (CMR) imaging is the gold standard to assess myocardial fibrosis and function. However, cardiac catheterization is invasive, and the contrast agents in LGE CMR are contraindicated for patients with end-stage renal disease, making them of great limitation in clinical application. Global diastolic strain rate index derived by 2D-STI is confirmed to have a strong correlation with myocardial fibrosis defined by LGE CMR and haemodynamic indices (Ʈ and LVEDP) both in patients and in animal models, it is less angle and load dependent and not influenced by valvular pathology [23]. And the guideline also indicated this novel parameter has been used in conjunction with mitral E velocity to estimate LV filling pressures and to predict outcomes in several disease states [10]. So global diastolic strain rate index reveals higher accuracy in diagnosing diastolic dysfunction and the degree of myocardial fibrosis in PD patients [5, 24–27].

In our study, we found that diastolic strain rate index in young PD patients is significantly different as compared to the age- and sex-matched controls, and the severity of diastolic dysfunction was associated both with low diastolic strain rate index, as other studies previously reported [6, 28]. Combined with E, E/DSrE still had a significant difference, confirming that LV diastolic dysfunction does exist at the early stage of peritoneal dialysis in young uremic patients.

We also found that there was a significant difference in DSrA, and E/DSrA between the two groups, which is in accordance with the known idea that LA function is closely coupled to LV diastolic function, and the increase in LA function is thought to be a mechanism counterbalancing the progression of LV diastolic dysfunction [7, 29]. In the presence of normal LA pressure, this shifts a greater proportion of LV filling to late diastole after atrial contraction in order to maintain the early filling pressures. From another point of view, the decrease in the ratio of E/DSrA may suggest that potential diastolic dysfunction may exist in young PD patients at the early stage [30].

By using stepwise multivariate linear regression, we estimated the association between diastolic strain rate index and conventional echocardiography parameters. We found that DSrE has a strong relationship with LVPWT and E/A. Considering the limitations of E/A, such as E/A ratio is age dependent, and the U-shaped relation between E/A and LV diastolic function may make it difficult to differentiate normal pseudonormal filling, particularly with normal LVEF [10], leading to E/A unsuitable for our case. Our study suggested that for clinical purposes, when patients are not yet diagnosed with LV diastolic dysfunction by conventional echocardiography suggested by guideline, the increase in LV wall thickness should be considered as risk factors. Previous studies have confirmed the important clinical significance of LVPWT, it is an independent risk factor for paroxysmal atrial fibrillation [31], and is significantly higher than those with dipper hypertension [32], and also highly reproducible measurements used as part of a multimodality approach when assessing morphologic LV preparedness in patients with CCTGA undergoing anatomic repair [33]. AS we know, LV hypertrophy can occur at the early stage of end-stage renal disease, so we highly recommended that the combined assessment of the DSrE and LVPWT should be taken into account to evaluate abnormal LV diastolic function at the early stage [30].

Our study had its strengths. First, this was an original study performed by a group of researchers who were one of the first to focus on LV diastolic function in young PD patients. Second, we used novel and noninvasive measurements to evaluate LV diastolic dysfunction that may be detected in a preclinical phase. Third, we elucidated the possible relationship between the strain rate and conventional echocardiography parameters, making it possible and more reliable to predict the LV diastolic dysfunction by observing conventional echocardiography parameters. One limitation of our study was the small sample size, and that it was only a single-center observational and cross-sectional study. Our study subsequently did not have the hemodynamic parameters obtained by cardiac catheterization as standards to compare data. And how much of diastolic abnormalities were due to reduced GLS avg. versus intrinsic diastolic dysfunction was difficult to ascertain. Moreover, our study just simply provided a sensitive tool to early diagnosis of diastolic dysfunction.

## Conclusion

Despite the preserved LVEF and so-called normal diastolic function parameters that are revealed by conventional echocardiography, it was determined that young PD patients had already developed diastolic dysfunction compared with the controls. Diastolic strain rate index based on 2D-STI could be used to evaluate early LV diastolic function, and multi parameter analysis including LVPWT should be taken into account when predicting the LV diastolic dysfunction in clinical practice. If some of them indicated abnormality, preventative measures should be implemented to diminish diastolic dysfunction and thus mortality and cardiovascular events.

## Data Availability

The datasets generated during the current study are not publicly available due to our research is being followed up. Our study has been registered to the Chinese Clinical Trial Registry (NO. ChiCTR1900024999), [http://www.medresman.org], the raw data supporting our findings will be available until the end of our study.

## Abbreviations

2D-STI: Two-dimensional speckle tracking imaging
A: Late diastolic velocity (by Pulsed Doppler)
a’: Peak velocity of the late diastolic wave (by Pulsed-wave tissue Doppler)
BMI: Body mass index
BSA: Body surface area
BUN: Blood urea nitrogen
Ca: Calcium
CKD: Chronic kidney disease
CMR: Cardiac magnetic resonance
CREA: Serum creatinine
DBP: Diastolic blood pressure
DSr avg.: Average LV global longitudinal diastolic strain rate
DSrA: DSr during late diastole
DSrE: DSr during early diastole
DSrIVR: DSr during isovolumic relaxation period
DT: Deceleration time of the E-wave (by pulsed Doppler)
E: Peak early diastolic velocity (by Pulsed Doppler)
e’: Peak velocity of the early diastolic wave (by Pulsed-wave tissue Doppler)
ESRD: End stage renal disease
GLS avg.: Average LV global longitudinal systolic strain
Hb: Hemoglobin
IVST: Interventricular septal thickness
LAVI: Left atrial maximum volume index
LGE: Late gadolinium enhancement
LV: Left ventricular
LVEDP: LV end-diastolic pressure
LVEF: LV ejection fraction
LVIDD: Left ventricular internal diameter at end-diastole
LVIDS: Left ventricular internal diameter at end-systole
LVMI: LV mass index
LVPWT: LV posterior wall thickness
P: Phosphorus
PD: Peritoneal dialysis
RAAS: Renin-angiotensin-aldosterone system
SBP: Systolic blood pressure
TR: Peak velocity of tricuspid regurgitation (by Continuous-wave Doppler)
